# Quantitative High Density EEG Brain Connectivity Evaluation in Parkinson’s Disease: The Phase Locking Value (PLV)

**DOI:** 10.3390/jcm12041450

**Published:** 2023-02-11

**Authors:** Lazzaro di Biase, Lorenzo Ricci, Maria Letizia Caminiti, Pasquale Maria Pecoraro, Simona Paola Carbone, Vincenzo Di Lazzaro

**Affiliations:** 1 Unit of Neurology, Neurophysiology, Neurobiology and Psichiatry, Department of Medicine and Surgery, Università Campus Bio-Medico di Roma, Via Alvaro del Portillo, 21, 00128 Rome, Italy; 2 Neurology Unit, Fondazione Policlinico Universitario Campus Bio-Medico, Via Alvaro del Portillo, 200, 00128 Rome, Italy; 3 Brain Innovations Lab, Università Campus Bio-Medico di Roma, Via Álvaro del Portillo 21, 00128 Rome, Italy

**Keywords:** quantitative EEG analysis, high density EEG, brain connectivity, phase locking value (PLV), Parkinson’s disease, biomarkers

## Abstract

Introduction: The present study explores brain connectivity in Parkinson’s disease (PD) and in age matched healthy controls (HC), using quantitative EEG analysis, at rest and during a motor tasks. We also evaluated the diagnostic performance of the phase locking value (PLV), a measure of functional connectivity, in differentiating PD patients from HCs. Methods: High-density, 64-channels, EEG data from 26 PD patients and 13 HC were analyzed. EEG signals were recorded at rest and during a motor task. Phase locking value (PLV), as a measure of functional connectivity, was evaluated for each group in a resting state and during a motor task for the following frequency bands: (i) delta: 2–4 Hz; (ii) theta: 5–7 Hz; (iii) alpha: 8–12 Hz; beta: 13–29 Hz; and gamma: 30–60 Hz. The diagnostic performance in PD vs. HC discrimination was evaluated. Results: Results showed no significant differences in PLV connectivity between the two groups during the resting state, but a higher PLV connectivity in the delta band during the motor task, in HC compared to PD. Comparing the resting state versus the motor task for each group, only HCs showed a higher PLV connectivity in the delta band during motor task. A ROC curve analysis for HC vs. PD discrimination, showed an area under the ROC curve (AUC) of 0.75, a sensitivity of 100%, and a negative predictive value (NPV) of 100%. Conclusions: The present study evaluated the brain connectivity through quantitative EEG analysis in Parkinson’s disease versus healthy controls, showing a higher PLV connectivity in the delta band during the motor task, in HC compared to PD. This neurophysiology biomarkers showed the potentiality to be explored in future studies as a potential screening biomarker for PD patients.

## 1. Introduction

The diagnosis of Parkinson’s disease (PD) is currently based on the clinical evaluation Poewe, et al. [1] of the cardinal motor symptoms, bradykinesia, rest tremor, and rigidity, which represent the hallmarks for the in vivo diagnosis [2] according to the current diagnostic criteria for PD [3]. Different strategies have been explored to characterize PD features in a non-invasive way. One first approach is to follow the clinical diagnostic pathway trying to make clinical evaluations of motor symptoms more objective and quantitative, through a motion analysis technique able to characterize PD motor symptoms [4,5,6], such as bradykinesia [7,8,9], tremors [10,11,12,13], rigidity [9,14,15,16], and axial symptoms, such as gait, balance, and postural issues [17,18,19,20,21,22], also with the support of machine learning algorithms [23,24,25,26,27]. Another possible approach is to explore the brain activities that underly and determine the PD symptoms, which are characterized by pathological oscillatory activities [28,29] and have been widely used to manage therapy, such as deep brain stimulation [30,31], but can be used also as a proxy for PD neurophysiology biomarkers identification.

In this context, neurophysiological tests may help to better understand the pathophysiology of PD, and their low cost, brief execution times, and the wide diffusion among hospitals represent a competitive advantage in respect to other techniques to support PD biomarkers identification in clinical practice.

Brain connectivity is a method to explore the way how different brain regions interact and communicate with each other. The degeneration of nigrostriatal dopaminergic neurons, which is the hallmark of the pathophysiology of PD, leads to the dysfunction of the basal ganglia–thalamo-cortical pathway, which underlies the PD motor symptoms [32].

Resting state functional MRI (RS-fMRI) can be used to study the connectivity among different brain areas in PD patients. A meta-analysis of RS-fMRI connectivity studies in PD patients [33], showed a decreased functional connectivity within the posterior putamen. The functional network involving this area and its cortical projections can be modulated by levodopa administration [32,33,34].

Among the neurophysiological techniques, electroencephalogram (EEG) is one of the most versatile and widely available techniques, it offers good balance between the temporal and spatial resolution, meaning that this technology is most frequently used in studies on PD biomarkers.

In de novo PD patients, compared to controls, a reduced coherence in α-β EEG frequency bands and a hyperconnectivity in γ band were observed [35].

Exploring dynamic networks between neuronal populations in a quantitative way, by noninvasive electrophysiological mapping with EEG, could unveil crucial information about brain connectivity in PD and subsequently, improve the diagnostic process.

Nonlinear and nonstationary systems may be analyzed with the phase locking methodology [36]. Indeed, the brain can be compared to a nonlinear dynamic system and, as such, the phase locking approach can be used for the scope [36,37,38]. Phase locking value (PLV) is a non-linear measure of pairwise functional connectivity (Lee, Liu et al., 2019), used to quantify the phase coupling between two biological nonlinear signals in a time-series, such as electroencephalographic signals [39]. A high PLV between two brain regions indicates a high synchrony [40].

The present study aims at investigating brain connectivity, through quantitative EEG analysis in Parkinson’s disease versus healthy controls, at rest and during a motor task, exploring the performance of the phase locking value (PLV) in discriminating the two study groups.

## 2. Methods

### 2.1. Patients and Data Collection

The database and EEG data utilized in this study were obtained from the University of Iowa Hospitals & Clinics (UIHC) Movement Disorders Clinics [41]. The database contains high-density EEG (HD-EEG) [42] data from 26 patients with PD and 13 demographically matched healthy controls (HCs). All patients in the experiment met the UK Parkinson’s Disease Brain Bank criteria for the diagnosis of idiopathic PD [43]. All patients underwent neuropsychological evaluation using the Montreal cognitive assessment (MOCA), EEG signals were recorded at rest and during a specific lower-limb pedaling motor task [41] using a customized 64-channels cap (EASYCAP GmbHAm Anger, 582237 Woerthsee-Etterschlag, Germany) with a high-pass filter of 0.1 Hz and a sampling rate of 500 Hz (Brain Products). Online reference and ground channels were Pz and FPz, respectively. Patients and HCs were both instructed to perform a lower-limb motor task during the EEG recording. Therefore, for each subject we analyzed the EEG recorded in both conditions (i.e., Resting State and Motor Task).

### 2.2. Quantitative EEG Analysis

Quantitative EEG analysis was performed using the Brainstorm Toolbox for MATLAB (Tadel et al., 2011) (The Math Works Inc., Natick, MA, USA), and in home MATLAB code. Offline data pre-processing was performed using Brainstorm and included: (i) DC removal; (ii) 60-Hz notch filter; (iii) bandpass filter between 1 and 70 Hz (linear phase finite impulse response filter); (iv) EEG re-reference to average; (v) and correction for pulse and eye-blink artifacts using independent component analysis [44,45].

### 2.3. EEG Connectivity Analysis

To assess the differences in brain networks among PD and HCs we performed a measure of EEG functional connectivity. We selected a total of 180 s continuous epoch from the EEG recordings free from relevant artifacts for further analysis [46,47]. As a measure of connectivity, we computed the phase locking value (PLV). PLV is an important measure of synchronization when studying bio-signals and especially electrical brain activities. It is a measure of non-directional frequency-specific synchronization reflecting long-range integrations and it assesses the extent to which the phase difference between two signals changes over time [36,37,48].

Taking into account the lack of consensus in the classification of frequency bands for quantitative EEG analysis [47], starting from the most recent International Pharmaco-EEG Society (IPEG; [49]) recommendations, also endorsed by the International Federation of Clinical Neurophysiology recommendations on frequency and a topographic analysis of resting state EEG rhythms [47], the final frequency band selected for the phase locking value connectivity analysis was based on the frequency band employed in several previous studies [45,46,48] in which, with respect to the IPEG recommendation, was selected the fastest delta band 2–4 Hz and a restricted theta band 5–7 Hz. We measured the PLV for all possible channel combinations and averaged to obtain a measure of global connectivity [46,48] for the following frequency bands: delta: 2–4 Hz; theta: 5–7 Hz; alpha: 8–12 Hz; beta: 13–29 Hz; and gamma: 30–60 Hz. Connectivity analysis was performed separately for the resting state EEG and for the EEG recorded during the lower limb pedaling motor task.

### 2.4. Statistical Analysis

Statistical analysis was performed using the R statistical package [50] and MATLAB (Mathworks). Data distribution was checked by means of a Kolmogorov–Smirnov test. The differences in Global Connectivity among PD and HCs was tested using a three-way aligned rank transformed (ART) ANOVA for non-parametric factorial three-way designs [51] with Frequency (five levels: delta, theta, alpha, beta, gamma), Group (two levels: PD and HCs) and Condition (two levels: resting state and motor task) as within the subject factor. A Bonferroni correction was used for post-hoc tests of multiple comparisons when needed.

To estimate the clinical value of EEG connectivity for differentiating between PD and HCs, we built receiver operating characteristic (ROC) curves on the PLV connectivity values for each frequency band and for each condition (i.e., resting state and motor task).

The following performance metrics were estimated in terms of outcome prediction: (i) sensitivity (ii) specificity, (iii) positive predictive value, (iv) negative predictive value; and (v) accuracy. The ROC curve point showing the highest combination of predictive values was selected as the optimum cut-off value to differentiate PD vs. HCs. Finally, we built non-parametric ROC curves to estimate 95% confidence intervals (CIs) for the area under the curve (AUC), sensitivity, specificity, positive predictive value (PPV), negative predictive value (NPV), and accuracy. CIs were validated using 10,000 stratified bootstrap replicates [52]. Moreover, a Spearman correlation test was used to assess the correlation between MOCA scores and the PLV in each frequency band. Significance level was set at *p* < 0.05. Results are reported as the mean ± standard deviation unless differently stated.

## 3. Results

### 3.1. Patient Cohort and Control Group

PD patients (nine females and 17 males) had a mean disease duration of 6.2 years (SD: ±3.7), a mean age of 67.3 years (SD: ±9.2), a UPDRS III score of 14.8 (SD: ±7.1), and a MOCA score of 23.3 (SD: ±3.9). The healthy controls (five females and eight males) had a mean age of 68.9 years (SD: ±8.2) [41].

### 3.2. Comparison between PD and Control Groups

#### 3.2.1. EEG Connectivity

**Figure 1:**
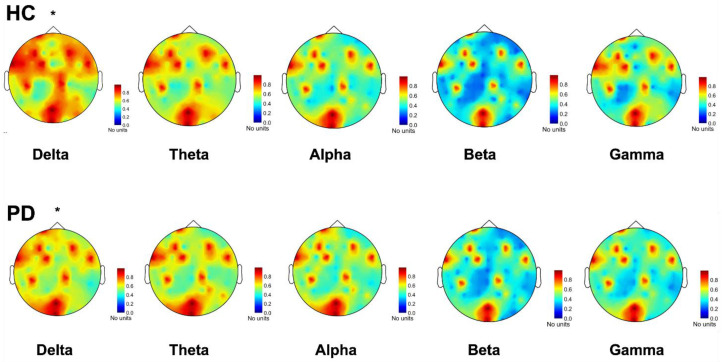
Phase locking value (PLV) connectivity topoplot and comparison between Parkinson Disease (PD) and Healthy Control (HC). PLV is expressed as the average across channels to obtain a measure of global connectivity. Notice how PLV in the delta range is higher in HC compared to PD. *: *p* < 0.05.

**Figure 2:**
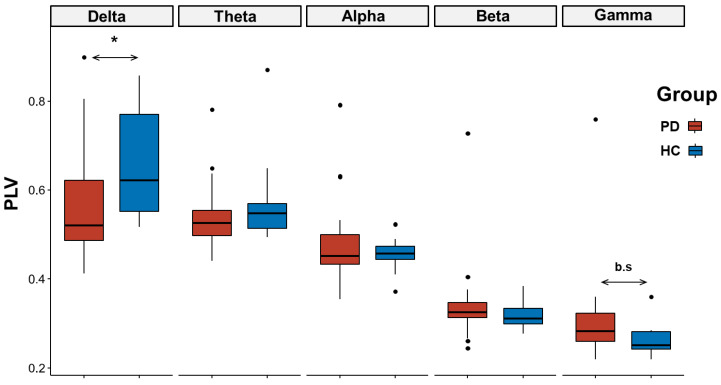
Boxplot distribution of the phase locking value (PLV) connectivity values between Parkinson disease (PD, red) and healthy control (HC, blue) across different frequency bands during the motor task. Black lines represent median values. Dots denote values that are farther than 1.5 interquartile ranges. Notice how PD subjects present a lower delta connectivity (*p* = 0.04) and a higher gamma connectivity, although with a borderline level of significance (*p* = 0.05). *: *p* < 0.05.

The comparison between PD and HCs revealed no significant differences between groups (factor *group*: F_(1,370)_ = 0.76, *p* = 0.38), but a significant group by frequency interaction (F_(4,370)_ = 3.62, *p* < 0.005; Figure 1), related to a higher connectivity in the delta frequency band for HCs compared to PD (Bonferroni corrected *p* = 0.04; Figure 2). We also found lower connectivity values in the gamma frequency band for HCs compared to PD, although with a borderline level of significance (Bonferroni corrected *p* = 0.05; Figure 2).

**Figure 3:**
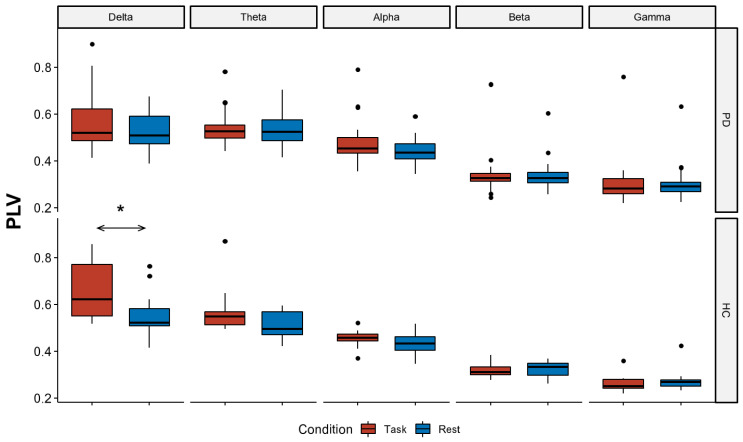
Boxplot distributions of the phase locking value (PLV) mean connectivity values. Boxplot distributions of the mean PLV values for different frequency bands across Groups: Parkinson disease (PD) vs. healthy control (HC) and conditions: motor task (red) vs. resting state (blue). Black lines represent median values. Dots denote values that are farther than 1.5 interquartile ranges. Connectivity values were significantly higher during the motor task compared to the resting state in HC (*p* = 0.004), as opposed to PD (*p* = 0.18). *: *p* < 0.05.

The ART ANOVA considering *condition* and *frequency,* as within the subject factor, showed a significant *condition* effect (F_(1,370)_ = 10.77, *p* = 0.001), related to higher global connectivity values during the motor task compared to the resting state. A significant *group* by *condition* interaction was also found (F_(1,370)_ = 5.33, *p* = 0.02). Post-hoc tests revealed a significant difference in connectivity values during the motor task compared to the resting state in HCs (Bonferroni corrected *p* = 0.004; Figure 3), as opposed to PD patients who did not reach the statistical significance (*p* = 0.18). We also found a significant *condition* by *frequency* interaction (F_(4,370)_ = 3.48, *p* = 0.008; Figure 3), related to higher delta connectivity values during the motor task, as opposed to the resting state (Bonferroni corrected *p* = 0.03; see Figure 3). Finally, we found no correlation between the PLV connectivity values and MOCA scores in each frequency band (*p* > 0.05).

#### 3.2.2. ROC Curve Analysis

**Figure 4:**
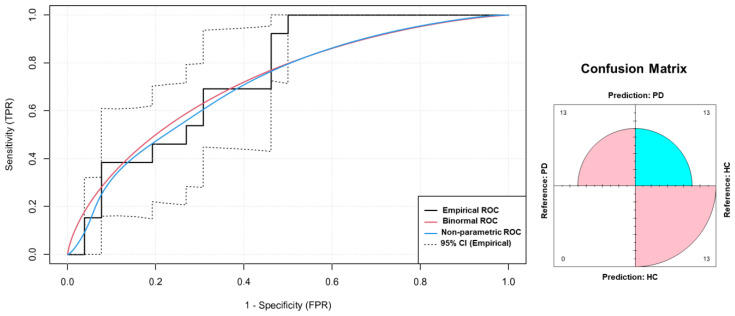
Receiver operating characteristic (ROC) curve (black line) (left image) and confusion matrix (right image) of the phase locking value (PLV) in the delta frequency band during the motor task for the classification of healthy controls (HCs) and Parkinson disease (PD) patients in our cohort. Non-parametric ROC curve (blue line), binormal ROC curve (red line) and 95% confidence interval (C.I.; dotted lines) are shown. AUC = area under the curve. CI = confidence interval. TPR = true positive ratio; FPR = false positive ratio.

The ROC curve analysis showed that the PLV connectivity analysis in the delta frequency band during the motor task band was able to differentiate HC from PD (Figure 4) with an area under the curve (AUC) of 0.75 (95% CI, 0.58–0.89), a sensitivity of 100% (95% CI, 100–100%), a specificity of 50% (95% CI, 31–69%), a PPV of 50% (95% CI, 42–62%), an NPV of 100% (95% CI, 100–100%), and an accuracy of 66.7% (95% CI, 54–79%).

## 4. Discussion

In the present study, we evaluated brain connectivity through a quantitative EEG analysis in Parkinson’s disease versus healthy controls, at rest and during a pedaling motor task, exploring the diagnostic performance of the phase locking value (PLV) in discriminating the two study groups.

In the literature, few studies explored the PLV analysis in the PD population. Bertrand, McIntosh, Postuma, Kovacevic, Latreille, Panisset, Chouinard and Gagnon [40] compared the baseline resting state EEG of healthy subjects and PD patients, and after a follow-up classified the PD patients who developed dementia and patients who did not developed dementia. The results were assessed in terms of both signal synchrony and variability at different timescales, respectively, and statistically expressed by the PLV and multiscale entropy (MSE). In the delta frequencies, the PLV was lower in the PD who developed dementia compared to the PD without dementia and controls, while, for the beta and gamma frequencies, the PD-dementia patients showed a higher PLV when compared with the PD-non dementia patients, and both groups showed a higher PLV when compared to the controls. Conversely, the signal variability was lower at the higher frequencies and higher at the lower ones.

The main hypothesis in Gerardo Sánchez-Dinorín et al.’s [53] research was that functional connectivity abnormalities could predict cognitive decline in Parkinson’s disease. The study showed that the increased synchrony of frontal slow waves predicts cognitive decline in PD patients after less than a decade with the illness [53].

In Soojin Lee et al.’s [54] study, the PLV was employed to evaluate the effect of dopaminergic medication and electrical vestibular stimulation (EVS) in Parkinson’s disease. While levodopa medication was effective in normalizing the mean PLV only, all EVS stimuli normalized the mean, variability, and entropy of the PLV in the PD subject, demonstrating both low- and high-frequency EVS exert widespread influences on cortico-cortical connectivity [54].

In the present study, the results showed no significant differences in the PLV connectivity between the two groups (PD vs. HCs) during the resting state, but a higher PLV connectivity in the delta band during the motor task in the HCs compared to PD. In addition, comparing the resting state versus motor task for each group, only in the HC results showed a higher PLV connectivity in the delta band during the motor task. These results showed a deficit for the PD subjects in modulating the delta band PLV brain synchrony during movement, in contrast with the healthy controls. In addition, in our study the PLV connectivity was not correlated with cognitive performance.

These preliminary results also show that the higher value of the PLV during the motor task could be a potential useful tool as a neurophysiological connectivity biomarker for PD. Considering the ROC AUC of 0.75, which indicates a good discrimination performance, the sensitivity of 100%, indicating the ability to identify a high number of patients potentially affected by PD, and a NPV of 100% indicating the ability to exclude only truly HCs, combined with its lower specificity and PPV, leads this predictor to be the candidate as a screening biomarker.

The main limitations of the study are the small number of the sample, the type of motor task which was not compared to different motor tasks of lower limbs or tasks of upper limbs, and in line with the lack of consensus in the classification of frequency bands for the quantitative EEG analysis [47], the specific band selected for the present study can be a limitation, therefore further studies are needed to confirm the results and the proposed applications.

## 5. Conclusions

The present study evaluated the brain connectivity through a quantitative EEG analysis in Parkinson’s disease versus healthy controls, showing a higher PLV connectivity in the delta band during the motor task in the HCs compared to PD. This neurophysiology biomarker showed the potentiality to be explored in future studies as a potential screening biomarker for PD patients.

## Data Availability

The data presented in this study are openly available in http://www.predictsite.com/ (accessed on 1 January 2022) [42].
